# The Impact of Cooperative Social Organization on Reducing the Prevalence of Malaria and Intestinal Parasite Infections in Awramba, a Rural Community in South Gondar, Ethiopia

**DOI:** 10.1155/2014/378780

**Published:** 2014-08-11

**Authors:** Gebeyehu Yihenew, Haileeyesus Adamu, Beyene Petros

**Affiliations:** ^1^Department of Biology, Addis Ababa University, P.O. Box 1176, Addis Ababa, Ethiopia; ^2^Institute of Biotechnology, Addis Ababa University, . P.O. Box 42524, Addis Ababa, Ethiopia; ^3^Department of Microbial, Cellular and Molecular Biology, College of Natural Sciences, Addis Ababa University, P.O. Box 1176, Addis Ababa, Ethiopia

## Abstract

*Introduction.* Parasitic diseases are the major causes of human health problem in Ethiopia. The high prevalence of parasitic infections is closely correlated with poverty, poor environmental hygiene, and impoverished health services. *Objective.* The study was conducted to assess the impact of health-conscious Awramba cooperative community and its neighboring communities on the prevalence of parasitic infections in South Gondar, Ethiopia. *Methods.* Single stool specimens were collected from 392 individuals from Awramba and the neighboring communities. Specimens were examined microscopically for the presence of parasites using microscopy. Questionnaire was administered to determine the knowledge attitude and practice (KAP) of study participants. *Results.* Of the total 392 study participants examined, 58(14.8%) were positive for malaria and 173 (44.1%) for intestinal parasites. The prevalence of malaria in Awramba community (5.1%) was less than that in neighboring communities (24.5%). The prevalence of parasitic infections in Awramba (18.8%) was less than that of the neighboring communities (69.4%). *Conclusion.* This study showed that good household and environmental hygiene, good toilet construction and usage, and proper utilization of ITN in Awramba cooperative community have significantly contributed to the reduction of the burden of parasitic infections. Thus, the positive achievement in reducing parasitic infections in Awramba cooperative community could be used as a model for affordable health intervention in the neighboring communities, in particular, and the whole country in general.

## 1. Introduction

Parasitic diseases caused by helminths and protozoa are the major causes of human and animal health problems in most underdeveloped countries including Ethiopia. The high prevalence of parasitic infections is closely correlated with poverty, poor environmental hygiene, and impoverished health services [1]. Malaria is the leading cause of morbidity in Ethiopia, with more than one million clinical cases reported annually [2].

Intestinal parasitic infections (IPIs) are globally endemic and have been described as constituting the greatest single worldwide cause of illness and disease [3, 4]. IPIs are linked to the lack of sanitation, lack of access to safe water, and improper hygiene, thus occurring wherever there is poverty [4, 5]. Intestinal parasitic infections, as in many developing countries, are common in Ethiopia and cause serious public health problems such as malnutrition, anemia, and growth retardation as well as higher susceptibility to other infections [6].

Many studies have addressed that lack of community awareness about the mode of transmission of parasites, and the unhygienic lifestyles of the communities contribute to the spread of parasitic diseases [7, 8].

## 2. Materials and Methods

### 2.1. Study Area

Awramba cooperative and its neighboring communities villages (specifically villages of: Arbachan, Qorke, Jib-Gudguad, Laydewol, Wojiterara, Maksegn, Tizaba, Timinda, Warsa, and Dej-Mesk). These communities are found in Wojiarbamba Kebele, Fogera Woreda, South Gondar Zone of Amhara National Regional State in Northwestern Ethiopia which is located 62 kilometers in the north of Bahir Dar. Wojiarbamba Kebele is divided into 27 local villages called “Gots.” The total population of Wojiarbamba Kebele was 8843 [9].

Awramba is a unique cooperative community, located in Turign “Got” in Wojiarbamba Kebele that diverts 5 km to the west inside from the main road of Bahir Dar city to Debre Tabor. This community is the brainchild of Zumra Nuru, a 62-year-old man, who is the founder and chair of Awramba cooperative community (founded in 1980). Currently, Awramba cooperative community has some 403 members in 109 households, living in over 17 hectares of land. The Awramba cooperative community is lauded in 2007 as a model to alleviate poverty and promote gender equality, model center for reproductive health, and area of best practices of leadership in a country where women generally hold a subservient status to men. As a result, in Awramba community, men cook, women plow, and gender difference has no place. The community is distinct in the fact that its members work together and are diligent, disciplined, and self-confident.

### 2.2. The Study Participants

A total of 392 study participants, from all age groups and both genders, were included in this study from the two communities with 196 study participants randomly sampled from each community. A simple random sampling method was employed in the selection of study participants in both study sites.

### 2.3. Specimen Collection and Examination

Fresh fecal samples were collected from the study participants in plastic vials labeled with identification numbers. A small portion of the fresh stool was microscopically examined in the field as a wet mount preparation for motile trophozoites and the remaining part was preserved in SAF (15 g sodium acetate, 20 mL glacial acetic acid, 40 mL formalin, and 925 mL distilled water) in the ratio of 1 g of stool to 3 mL of SAF for later examination and in the laboratory. The preserved stool samples were processed by formol-ether concentration method for parasitic eggs or cysts. Parts of the samples were processed by modified by Ziehl-Neelsen staining method for oocyst examination.

### 2.4. Blood Sample Collection and Examination

Finger prick blood samples were taken from each study participant and thick and thin blood spears prepared by following the standard procedure. The blood smears were stained with Giemsa stain. Thick and thin blood smears were examined for detection and species confirmation of malaria parasites, respectively.

### 2.5. Hemoglobin Test

The study participants were tested for anemia by using the HemoCue (Brand & Co., US) device.

### 2.6. Sociodemographic Data Collection

A structured questionnaire to assess the knowledge, attitude, and practice (KAP) of the study participants about parasitic diseases and their transmission was developed and administered to each study participant to obtain information on their sociodemographic characteristics.

### 2.7. Data Analysis

Influence of sex, age, KAP, and other factors on the prevalence of parasites in the two communities was analyzed using chi-square (*χ* ^2^) test at 95% level of significance. The statistical analyses were performed using the statistical software SPSS software version 17.

### 2.8. Ethical Consideration

This study was conducted with the approval of the Ethical Review Committee of Research, Department of Biology, Addis Ababa University. Informed written consent was taken from each study participant. Participants were also informed that they are free to withdraw consent at any time and their records and specimen will be examined by authorized persons, and all personal information on them will be treated as confidential. Clinicians managed those participants positive for intestinal parasites and malaria.

## 3. Results

Microscopic stool sample examination revealed an overall prevalence of 44.1% of intestinal parasite infection in the two communities. However, the prevalence of intestinal parasites in Awramba cooperative community was much lower (18.9%) than in the neighboring communities (69.4%) and the difference was statistically different (*P* < 0.05) (Table 1).

Overall, ten different intestinal parasites were detected in the two communities. They are* Ascaris lumbricoides* (9.4%),* Strongyloid stercoralis* (0.3%),* Enterobius vermicularis* (3.6%),* Schistosoma mansoni* (1.3%),* Cryptosporidium* spp. (1.8%), hookworm (11.2%),* Hymenolepis nana* (0.8%),* Giardia lamblia* (4.3%), and* Entamoeba histolytica/dispar* (8.7%). Furthermore, the highest level of intestinal parasite infection (24.7%) was recorded in the age group ≥15 years old (Table 2). The prevalence of intestinal protozoa in the age group of 6–14 years was 11.3% in Awramba cooperative community and 35.5% in neighboring communities. In addition, intestinal protozoa prevalence in the age group ≥15 years was 3.7% in Awramba and 24.3% in the neighboring communities. Similarly, the prevalence of intestinal helminths among the age group of 6–14 years was 8.2% in Awramba cooperative community and 58% in the neighboring communities and also significantly higher prevalence among the group of ≥15 years (47.8%) in the neighboring communities compared to that of Awramba (13.4%) was determined. The differences in parasite prevalence between the two communities were statistically significant (*P* < 0.05) (Table 2).

Blood smear examination of samples for malaria infection detected 5.1% positivity for Awramba and 24.5% for the neighboring communities. This showed malaria prevalence in neighboring communities to be significantly (*P* < 0.05) higher than the prevalence in Awramba.

The mean serum hemoglobin concentration, as measured with HemoCue analyzer, was 13.34 ± 2.03 g/dL among Awramba study participants and 12.6 ± 2.36 g/dL among those in the neighboring communities. The prevalence of anemia among Awramba (9.4%) study participants was significantly (*P* < 0.05) lower than that in the neighboring communities (13.8%) (Table 3).

Based on the KAP survey, malaria was mentioned as a health problem by 125 (63.8%) of Awramba study participants and 158 (80.6%) of the neighboring community study participants (Table 4) and the difference between the two proportions was significant (*P* < 0.05). The KAP study also revealed that a high proportion (30.1%) of the study participants from the neighboring communities never wore shoes compared to those from Awramba community (1%).

Study participants' responses about the hand washing habit before meal and after toilet showed 83.7% positive response from Awramba community and 58.2% from the neighboring communities (Table 4). On the other hand, significant difference (*P* < 0.05) was seen in consuming vegetables without proper washing and cooking among Awramba community (49.5%) and neighboring community (68.4%).

## 4. Discussion

From the findings of the KAP study, it can be deduced that Awramba cooperative community has high level of knowledge about the modes of parasitic disease transmission because they pay adequate attention to health education. This appears to have paid off in terms of better use of the various available disease control measures, such as proper ITN use and environmental management in malaria control; construction and use of latrines for domestic waste disposal; care for personal and food hygiene; protection from soil transmitted parasitic infections through food hygiene and wearing shoes [10]. Similar study in Nepal had shown the association of knowledge of unhygienic green vegetable consumption and intestinal parasite prevalence [11]. Also in a study from Ghana the very common consumption of unhygienic fresh vegetables among food vendors was shown to increase the risk of intestinal parasite infection in the population [12].

The higher prevalence of malaria among the study population of Awramba neighboring communities (24.5%) compared to the reports of the National Malaria Indicator Survey (4%) (10) and the findings in Oromia and SNNPR regions (2.4%) [13] and that of the Amhara Region (4.6%) [14] can be an indication of these communities being a malaria transmission “hotspot.” However, the malaria prevalence in Awramba community (5.1%) was not much different from the overall prevalence in the Amhara Region, implying that the Awramba cooperative community may have been benefiting from the overall health education efforts of the region. The high overall prevalence of malaria in the Awramba neighboring communities indicates that the burden of malaria is still high in different parts of the country as also reported from south west Ethiopia (10.5%). This is contrary to the dramatic decrease in malaria prevalence and the modeling on “trends of health and health related indicators” predicted over the last decade [15]. The local variation in malaria prevalence in Ethiopia may be influenced by focal antimalaria measures documented in this study whereby the prevalence was significantly higher in the neighboring communities compared to Awramba cooperative community, which, as revealed from the KAP findings, practices health-conscious environmental health measures.

The relatively lower prevalence of intestinal parasites in the health-conscious Awramba cooperative community (18.9%) compared to the neighboring communities (69.4%) is a good indication that health-conscious community measures, in addition to geographic and climatic variations, would significantly influence the prevalence of intestinal parasites. This can be seen from the high prevalence of these parasites from other parts of the country where that lack of health-conscious cooperative community exists—82.7% from villages in southwestern Ethiopia [14]; 65% among indigenous people in three resettlement farms of western Ethiopia [16]; and 61% from a small farming village near Lake Tana [17].

The prevalence of anemia in Awramba community (18.9%) was significantly lower than in the neighboring communities (27.6%). It was even lower than that reported from several other regions in Ethiopia: 34.2% from southwest [13], 47.2% from north Ethiopia [18], 47% from Oromia [19], and 42% from Tigray, is an indicator of health-conscious of these communities that practiced sanitation, malaria control, and food hygiene would have an improved health status [18] in this community in Ethiopia.

Although an iron deficient diet is known to be an important cause of anemia [19], it is unlikely that this is true to the Awramba neighboring communities since the population's major staple diet is “*teff*” (*Eragrostis teff*), a cereal which has high iron content mainly due to contamination with the soil surface over which it is harvested [20]. Therefore, since iron deficient diet cannot be taken as a primary cause of high anemia prevalence, the multiple effects of the high burden of parasitic infections (malaria and intestinal parasites) may have contributed to high prevalence of anemia in the Awramba neighboring communities that do not lead a health-conscious community life [21].

## 5. Conclusion

Significantly higher prevalence of malaria and intestinal parasites was observed in the neighboring communities as compared to Awramba cooperative community. The increased prevalence of intestinal parasites in the Awramba neighboring communities was associated with factors such as lack of awareness about modes of parasite transmission and indiscriminate defecation by the inhabitants and absence of well-constructed toilets, poor sanitation, and poor personal hygiene. Anemia was more prevalent in the Awramba neighboring communities and its level of prevalence was associated with parasite prevalence in the two communities. The study has presented some evidence to show that Awramba cooperative community's better KAP about the cause, mode of transmission, and prevention of malaria as well as intestinal parasite infections has protected the population from these health hazards. Therefore, in light of the positive health impact the Awramba cooperative community lifestyle has provided, conditions must be facilitated for the neighboring communities to share experience with Awramba community on personal and environmental hygiene, toilet construction and usage, and the relationship of the community to health extension workers. Health workers should mobilize the neighboring communities to improve the health situation through health education related to personal and environmental hygiene. It must also be stated that the Awramba cooperative community achievements in health improvement provide useful lessons of national relevance in the effort to control the neglected tropical diseases of the poor.

## Figures and Tables

**Table 1:** Prevalence of intestinal parasites among Awramba and its neighboring communities in Fogera Woreda, South Gondar Zone, Ethiopia (November 2010–April 2011).

Types of parasites	Awramba community (*n* = 196)			Neighboring communities (*n* = 196)			Total *n* = 392 Number observed (%)
	Male *n* = 105 Number observed (%)	Female *n* = 91 Number observed (%)	Total *n* = 196 Number observed (%)	Male *n* = 108 Number observed (%)	Female *n* = 88 Number observed (%)	Total *n* = 196 Number observed (%)	
Protozoa	9 (8.6)	5 (5.5)	14 (7.1)	23 (21.3)	21 (23.9)	44 (22.5)	58 (14.8)
* E. histolytica/dispar *	4 (3.8)	1 (1.1)	5 (2.6)	16 (14.8)	13 (14.8)	29 (14.8)	34 (8.7)
* Giardia lamblia *	3 (2.8)	1 (1.1)	4 (2)	7 (6.5)	6 (6.8)	13 (6.6)	17 (4.3)
* Cryptosporidium *spp.	2 (1.9)	3 (3.3)	5 (2.6)	—	2 (2.3)	2 (1)	7 (1.8)
Helminths	11 (10.5)	12 (13.2)	23 (11.8)	52 (48.1)	40 (45.5)	92 (46.9)	115 (29.3)
* A. lumbricoides *	1 (0.9)	7 (7.7)	8 (4.1)	14 (12.9)	15 (17)	29 (14.8)	37 (9.4)
* T. trichiura *	1 (0.9)	—	1 (0.5)	6 (5.5)	4 (4.5)	10 (5.1)	11 (2.8)
Hookworm species	7 (6.7)	3 (3.3)	10 (5.1)	19 (17.6)	15 (17)	34 (17.3)	44 (11.2)
* S. stercoralis *	1 (0.9)	—	1 (0.5)	—	—	—	1 (0.3)
* S. mansoni *	—	—	—	4 (3.7)	1 (1.1)	5 (2.6)	5 (1.3)
*E. vermicularis *	1 (0.9)	2 (2.2)	3 (1.5)	8 (7.4)	3 (3.4)	11 (5.6)	14 (3.6)
*H. nana *	—	—	—	1 (0.9)	2 (2.3)	3 (1.3)	3 (0.8)

Overall total	20 (19)	17 (18.7)	37 (18.9)	75 (69.4)	61 (69.3)	136 (69.4)	173 (44.1)

**Table 2:** Prevalence of intestinal helminth and protozoan infections among age groups of study participants in Awramba and its neighboring communities, Fogera Woreda, South Gondar Zone, Ethiopia (November 2010–April 2011).

Age group (yrs)	Study community	Number of participants	Intestinal protozoa		Intestinal helminths	
			Prevalence *n* (%)	*P* value	Prevalence *n* (%)	*P* value
1–5	Awramba	17	—	0.18	4 (23.5)	0.28
	Neighboring	50	5 (10)		19 (38)	

6–14	Awramba	97	11 (11.3)	0.01∗	8 (8.2)	0.00∗
	Neighboring	31	11 (35.5)		18 (58)	

≥15	Awramba	82	3 (3.7)	0.00∗	11 (13.4)	0.00∗
	Neighboring	115	28 (24.3)		55 (47.8)	

Total		392	58 (14.8)		115 (29.3)	

∗Significant difference (*P* < 0.05).

*P* value: comparing prevalence of parasites between the two study communities.

**Table 3:** Prevalence of anemia among different age groups of Awramba and its neighboring communities in Fogera Woreda, South Gondar Zone, Ethiopia (November 2010–April 2011).

Age group (years)	Study communities	Participants examined	Anemic study participants *n* (%)	*P* value
1–5	Awramba	17	4 (23.5)	(0.61)
	Neighboring	50	15 (30.0)	
6–15	Awramba	99	21 (21.2)	(0.05)
	Neighboring	31	12 (38.7)	
>15	Awramba	80	12 (15)	(0.15)
	Neighboring	115	27 (23.4)	

Total	Awramba	196	37 (18.9)	(0.04)∗
	Neighboring	196	54 (27.6)	

∗Significant difference (*P* < 0.05).

*P* value: comparing prevalence between the two study sites.

**Table 4:** Knowledge, attitude, and practice on malaria and intestinal parasites among Awramba and its neighboring communities in Fogera Woreda, South Gondar Zone, Ethiopia (November 2010–April 2011).

KAP about	Awramba community *n* = 196 *n* (%)	Neighboring communities *n* = 196 *n* (%)
Malaria as health problem	125 (63.8)	158 (80.6)
Mode of malaria transmission by mosquito bite	159 (81.1)	102 (52)
Stagnant water as a breeding site of mosquito	132 (67.3)	83 (42.3)
Protection of malaria vector by mosquito net	132 (67.3)	93 (47.4)
Consulting health centers when infected with malaria and intestinal parasites	168 (85.7)	145 (74)
Consulting traditional healers when infected with malaria and intestinal parasites	—	3 (1.5)
Using herbal remedies as malaria treatment	2 (1)	6 (3.1)
Malaria as a severe disease if not treated	158 (80.6)	80 (40.8)
The responsibility of malaria control is of all stakeholders	103 (52.6)	66 (33.7)
Disposing home garbage in open ground	12 (6.1)	102 (52)
Attending health education	115 (58.7)	75 (38.3)
Disposing home garbage by burning	149 (76)	38 (19.4)
Possessing toilet	175 (89.3)	72 (36.7)
Using toilet	158 (58.7)	43 (38.3)
Not wearing shoes	2 (1)	59 (30.1)
Washing hands before meal and after toilet routinely	164 (83.7)	115 (58.2)
Eating vegetables without proper washing and cooking	97 (49.5)	134 (68.4)
Eating raw meat	33 (16.8)	84 (42.8)
